# Evaluation of Electrodialysis Desalination Performance of Novel Bioinspired and Conventional Ion Exchange Membranes with Sodium Chloride Feed Solutions

**DOI:** 10.3390/membranes11030217

**Published:** 2021-03-19

**Authors:** AHM Golam Hyder, Brian A. Morales, Malynda A. Cappelle, Stephen J. Percival, Leo J. Small, Erik D. Spoerke, Susan B. Rempe, W. Shane Walker

**Affiliations:** 1 Center for Inland Desalination Systems (CIDS) and Nanotechnology Enabled Water Treatment (NEWT) Engineering Research Center, The University of Texas at El Paso, 500 W., University Ave., El Paso, TX 79968-0684, USA; bamorales3@miners.utep.edu (B.A.M.); macappelle@utep.edu (M.A.C.); 2 Sandia National Laboratories, Albuquerque, NM 87185-1315, USA; sperciv@sandia.gov (S.J.P.); ljsmall@sandia.gov (L.J.S.); edspoer@sandia.gov (E.D.S.); slrempe@sandia.gov (S.B.R.)

**Keywords:** electrodialysis, desalination, bioinspired, ion-exchange membrane, NaCl feed

## Abstract

Electrodialysis (ED) desalination performance of different conventional and laboratory-scale ion exchange membranes (IEMs) has been evaluated by many researchers, but most of these studies used their own sets of experimental parameters such as feed solution compositions and concentrations, superficial velocities of the process streams (diluate, concentrate, and electrode rinse), applied electrical voltages, and types of IEMs. Thus, direct comparison of ED desalination performance of different IEMs is virtually impossible. While the use of different conventional IEMs in ED has been reported, the use of bioinspired ion exchange membrane has not been reported yet. The goal of this study was to evaluate the ED desalination performance differences between novel laboratory‑scale bioinspired IEM and conventional IEMs by determining (i) limiting current density, (ii) current density, (iii) current efficiency, (iv) salinity reduction in diluate stream, (v) normalized specific energy consumption, and (vi) water flux by osmosis as a function of (a) initial concentration of NaCl feed solution (diluate and concentrate streams), (b) superficial velocity of feed solution, and (c) applied stack voltage per cell-pair of membranes. A laboratory‑scale single stage batch-recycle electrodialysis experimental apparatus was assembled with five cell‑pairs of IEMs with an active cross-sectional area of 7.84 cm^2^. In this study, seven combinations of IEMs (commercial and laboratory-made) were compared: (i) Neosepta AMX/CMX, (ii) PCA PCSA/PCSK, (iii) Fujifilm Type 1 AEM/CEM, (iv) SUEZ AR204SZRA/CR67HMR, (v) Ralex AMH-PES/CMH-PES, (vi) Neosepta AMX/Bare Polycarbonate membrane (Polycarb), and (vii) Neosepta AMX/Sandia novel bioinspired cation exchange membrane (SandiaCEM). ED desalination performance with the Sandia novel bioinspired cation exchange membrane (SandiaCEM) was found to be competitive with commercial Neosepta CMX cation exchange membrane.

## 1. Introduction

### 1.1. Background

Many studies of electrodialysis (ED) have evaluated the desalination performance of commercial and laboratory‑scale ion exchange membranes (IEMs), but most of these studies used independent sets of experimental parameters or conditions such as feed solution compositions and concentrations, superficial velocities of the process streams (diluate, concentrate, and electrode rinse), and applied electrical voltages [1,2,3,4,5,6,7,8,9,10,11,12]. IEMs work by allowing mainly ions to pass through them while rejecting the transport of water molecules (i.e., opposite of reverse osmosis or forward osmosis membranes). Most studies use only one type of anion exchange membrane (AEM) and cation exchange membrane (CEM) [1,2,3,4,5,6,7,10], and only a few compare two different types of AEM and CEM membranes [9,11,12]. Therefore, it is difficult to assess desalination performance of different commercially available and well-known IEMs and to recommend suitable IEMs for ED to achieve a desirable outcome (i.e., high ion‑selectivity, high salinity removal, high water recovery, low energy consumption, or low osmotic water flux). Moreover, the bioinspired IEM developed recently [13,14] has not been reported yet for use in ED.

### 1.2. Objectives

The goal of the study was to evaluate the ED desalination performance differences between novel laboratory‑scale bioinspired IEM and conventional IEMs, using a similar set of experimental parameters. The ED desalination performance of the IEMs was evaluated by determining (i) limiting current density, (ii) current density, (iii) current efficiency, (iv) salinity reduction in diluate stream, (v) normalized specific energy consumption, and (vi) water flux by osmosis as a function of (a) initial concentration of NaCl feed solution (diluate and concentrate streams), (b) superficial velocity of feed solution, and (c) applied stack voltage per cell-pair of membranes.

## 2. Materials and Methods

### 2.1. Experimental Plan and Variables

The laboratory-scale batch-recycle experimentation was planned to examine the pseudo-steady-state operation of the electrodialysis system as a function of time, which is equivalent to a full-scale single pass operation as a function of distance along the flow path, from inlet to outlet. The experimentation was designed, following another study [10], by maintaining dynamic similitude between full-scale and batch-recycle operation for (i) the velocity of the feed solution flow between AEMs and CEMs and (ii) the electric potential drop per cell-pair of membranes (consequently, the current density and current efficiency).

Discrete values and ranges of experimental variables are shown in Table 1. The feed water concentrations were representative of freshwater (1 g/L), brackish water (3–10 g/L), seawater (35 g/L), and produced water (100 g/L). The electrode rinse solution was prepared with a fixed concentration of 0.1 molar (14.2 g/L) sodium sulfate (Na_2_SO_4_). The range of electrical and hydraulic conditions simulated full-scale ED systems. The membranes used in this study were commercially available general desalination IEMs, commercial bare polycarbonate membrane (Polycarb), and laboratory-made Sandia novel bioinspired CEM (SandiaCEM). The ED desalination performance of seven membrane-pairs were compared through various experimental conditions (e.g., superficial velocity, stack voltage, and feed concentration) in terms of current density, current efficiency, salinity reduction in diluate stream, normalized specific energy consumption, and water flux.

### 2.2. Experimental System and Chemicals

**Figure 1:**
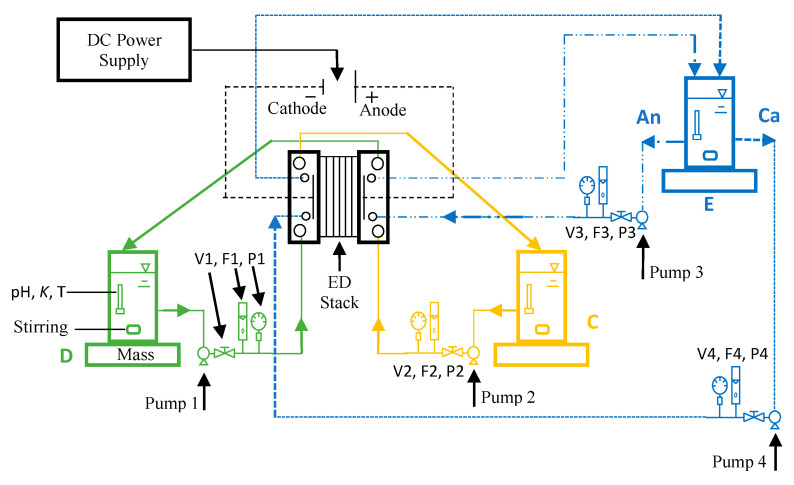
Schematic diagram for the batch-recycle electrodialysis system (adapted from Ref. [10]); *K* = conductivity, T = temperature; V = valve, F = flow meter, P = pressure gauge, D = diluate stream, C = concentrate stream, E = electrode rinse, An = anolyte, Ca = catholyte.

A batch-recycle electrodialysis experimental system was assembled, and a schematic diagram of the process is shown in Figure 1. A laboratory‑scale Master Flex peristaltic-cartridge pump (Cole-Parmer, Vernon Hills, IL, USA, Model: 7519-00) was used to circulate the solutions through each of the three process streams (i.e., diluate, concentrate, and electrode rinses). The flow rates through each of the process streams were controlled manually, and the flow rate was monitored manually at 30-min intervals. The process stream reservoirs were one-liter plastic bottles that are stirred by non-heating magnetic stirrers (Fisher Scientific, Waltham, MA, USA, model: Fisher 14-955-150). The electrical conductivity, pH, and temperature of the process stream reservoirs were determined using a pH/conductivity meter (Thermo Scientific, Bartlesville, OK, USA, model: Orion Star A325). The mass of the diluate reservoir was measured continuously to the nearest 0.1 g using a digital mass-balance (Meller Toledo, Columbus, OH, USA, model: XS2002S) to gravimetrically quantify the net mass of water and salt transportation across the membranes. Analog pressure gauges (Grainger low pressure gauge, Lake Forest, IL, USA, model: 18C774) were used at the inlet of the diluate, concentrate, and electrode rinse streams to observe the head loss through each stream and the average transmembrane pressures. A programmable DC Power Supply (B&K Precision, Yorba Linda, CA, USA, Model: 9123A) was used for monitoring and controlling voltage and current through the electrodialysis stack.

Laboratory-grade sodium chloride (NaCl, ACS reagent grade) and sodium sulfate (Na_2_SO_4_, ACS reagent grade) salts were purchased from Fisher Scientific (Waltham, MA, USA) to prepare the feed water and electrode rinse solutions, respectively. All reagent water was purified and deionized to a resistivity of 18.2 MΩ cm.

### 2.3. Electrodialysis (ED) Stack

A laboratory‑scale single stage electrodialysis stack (model: 08002-001) was purchased from PCCell/PCA, GmbH (Heusweiler, Germany). The anode was made of titanium metal with platinum/iridium coating, and the cathode was stainless steel. The end-plates, surrounding the electrodes and compressing the stack, were made of polypropylene. The active cross-sectional area of membrane subjected to the applied electric field was 7.84 cm^2^ (2.80 × 2.80 cm). Polyester mesh spacer-gaskets of thickness 0.45 mm physically separated the AEMs and CEMs. In assembling the ED stack, the end-plates compressing the stack were tightened until a given flow rate yielded the same pressure drops (i.e., 3 kPa) through the stack (i.e., from inlet to outlet of diluate and concentrate streams) for each experiment. For a specific combination of membranes, consistency across the replicate tests was confirmed by measuring the distance between the end-plates with a digital caliper.

### 2.4. Ion Exchange Membranes (IEMs)

In this study, seven combinations of IEMs (commercial and laboratory-made) were used:

- Neosepta AMX/CMX,
- PCA PCSA/PCSK,
- Fujifilm Type 1 AEM/CEM,
- SUEZ AR204SZRA/ CR67HMR,
- Ralex AMH-PES/ CMH‑PES,
- Neosepta AMX/Bare Polycarbonate membrane (Polycarb), and
- Neosepta AMX/Sandia novel bioinspired cation exchange membrane (SandiaCEM).

The Neosepta AMX/CMX membrane pair was considered as the control membrane to compare the performances of the other six membrane pairs with the AMX/CMX pair. Bare polycarbonate membrane (0.05 μm pore, 90 mm diameter, 6 μm thick) was purchased from Sterlitech Corporation (Kent, WA, USA). The detailed description of the development, prospects, applications, and properties of these commercial IEMs have been articulated in literature and the company’s websites [15,16,17,18,19,20,21,22,23,24,25,26]. However, a list of standard properties is summarized in Table 2.

A bioinspired membrane is a type of membrane that is developed to incorporate structural features of biological cellular membranes, specifically ion channel proteins [27]. Water permeable bioinspired desalination membranes using aquaporin (AQPs) water channels are reported for use in pressure-driven and osmotically-driven desalination [28,29,30,31,32].

However, the use of bioinspired IEM in ED has not been reported yet. To our knowledge, this is the first testing of a bioinspired IEM in electrodialysis. The Sandia novel bioinspired cation exchange membrane (SandiaCEM) used in this study was fabricated by Percival et al. [13,14] using a layer by layer (LbL) dip coating assembly process of electrolytes (e.g., polyacrylic acid (PAA) and polyethylimine (PEI)) with crosslinking reagents (e.g., glutaraldehyde (GA) and N-dimethylaminopropyl-N_0_-ethylcarbodimide hydrochloride (EDC)) over a bare polycarbonate (Polycarb) support membrane. The SandiaCEM demonstrated ionic selectivity, which was achieved with the use of LbL deposition of many nanostructured polyelectrolyte layers [13,14]. The cation transport selectivity was increased with the increasing number of polyelectrolyte layers when the polyelectrolyte or polymer layers were cross-linked with GA [13]. Ionic selectivity was independent of ionic conductivity, and the ionic conductivity was decreased with the coatings but was found to regain a portion of it upon crosslinking the polyelectrolyte [13,14]. Cross-linking the membranes also increased the intermolecular integrity of the polyelectrolyte films and inhibited the slow surface diffusion and redissolution of the polyelectrolyte films [13]. The SandiaCEM is an example of how a controllable and inexpensive method can be tailored to create ion-selective and chemically robust bioinspired membranes on porous supports for a wide range of applications.

### 2.5. Experimental Procedure

All membranes used in this study were soaked in 0.01 M NaCl solution for 24 h prior to use. Soaked membranes were trimmed to 6.4 × 4.4 cm size, and three holes were punched at precise locations along each side. The electrodialysis stack was assembled with five cell-pairs of cation- and anion-exchange membranes by arranging them in an alternating pattern between two electrodes. Five cell-pairs were built with five anion exchange membranes and five cation exchange membranes, and one additional Neosepta CMB membrane was always placed adjacent to the end spacer on the cathode side. The Neosepta CMB membrane is a cation exchange membrane that has high mechanical strength (burst strength ≥ 0.40 MPa) and alkali resistance (electrical areal resistance = 4.5 Ω cm^2^), and it can resist the effect of high pH occurring as a result of reduction of water on the cathode during the electrodialysis experiments [33].

Synthetic feed water solutions (diluate and concentrate) of 1, 3, 10, 35, and 100 g/L were prepared by adding NaCl salt in deionized water in the laboratory. The feed water concentrations were representative of freshwater (1 g/L), brackish water (3 and 10 g/L), seawater (35 g/L), and produced water (100 g/L). In the study, sodium chloride (NaCl) feed solution was used, but real brackish water, seawater, and produced water contains additional ions (e.g., calcium, sulfate, nitrate, and fluoride), depending on the sources water types and locations. Since the concentration of sodium and chloride ions are relatively abundant among brackish water, seawater, and produced water samples, a binary NaCl feed solution was used in this study [34]. As with this study, many other studies also used NaCl feed solution with different concentration ranges such as 3–40 g/L [34,35,36], even for 3–150 g/L [37]. Synthetic electrode rinse solution was also prepared by mixing a fixed concentration of 0.1 molar sodium sulfate (14.2 g/L Na_2_SO_4_) with deionized water in the laboratory.

The diluate solution was circulated at a flow rate of 15, 30, and 60 mL/min (corresponding to a superficial velocity of 2, 4, and 8 cm/s, respectively, through the diluate cells inside electrodialysis stack), and the pressure of the diluate cell for the relevant flow rate was recorded. The solution flow rate through the concentrate cells and electrode rinse compartments were adjusted to maintain the same pressure as diluate cells. The transmembrane pressure difference between the diluate, concentrate, and electrode rinse compartments was kept lower than 1.4 kPa (0.2 lb/in²), which was recommended by another study [25], to stabilize the electrodialysis system.

After stabilizing the flows and pressures, the voltage loss at the electrodes (including thermodynamic, overpotential, and ohmic contributions) was determined experimentally (see the calculation section of this article for more information). Afterward, the applied stack voltage (voltage requirement across the electrodialysis stack) was calculated by subtracting the voltage loss at the electrodes from the total applied voltage. Every 5 s, the experimental LabVIEW supervisory control and data acquisition (SCADA) system automatically calculated the voltage loss at the electrodes and recalculated the corresponding total applied voltage to maintain a desired stack voltage (e.g., 0.4, 0.8, or 1.2 V per cell-pair).

The experimental data for hydraulic (e.g., diluate reservoir mass change), electrical (e.g., applied voltage and current), and chemical (e.g., pH, temperature, conductivity) parameters were recorded automatically in excel spreadsheets in the computer by the LabVIEW SCADA system. Finally, the acquired data were analyzed to evaluate ED desalination performance of the novel laboratory‑scale bioinspired and conventional IEMs with respect to (i) current density, (ii) current efficiency, (iii) salinity reduction in diluate stream, (iv) normalized specific energy consumption, and (v) water flux by osmosis as a function of (a) initial concentration of NaCl feed solution (diluate and concentrate streams), (b) superficial velocity of feed solution, and (c) applied stack voltage per cell-pair of membranes. Each of the experiments was conducted in triplicate to check the accuracy and consistency of data while maintaining a standard deviation lower than 5%.

The step-by-step general experimental procedures are summarized below:

- Experimental and pre-rinse solutions (same as experimental concentration) were prepared for electrodialysis system equilibration.
- Pre-rinse solutions from the three process streams were circulated, and the experimental DC voltage was applied at the electrodes to approach the equilibration of the membranes with the solution.
- After evacuating the pre-rinse solution, the electrodialysis apparatus was loaded with the experimental solutions.
- The experiment was performed with full data acquisition.
- Acquired data were analyzed to determine ED desalination performance.

### 2.6. Data Acquisition and Control Hardware

A custom supervisory control and data acquisition (SCADA) system was developed in LabVIEW 2017 software for controlling and monitoring hardware such as the programmable mass-balance, DC Power supply, and pH/conductivity meters at five-second intervals during each of the experiment. The experimental data were recorded for hydraulic (e.g., flow rates and diluate reservoir mass change), electrical (e.g., applied voltage and current), and chemical (e.g., pH, temperature, conductivity) characterization.

### 2.7. Data Analysis

Output files generated by the custom LabVIEW SCADA system were saved automatically as spreadsheets, which were subsequently analyzed to calculate limiting current density, current density, charge efficiency, salinity reduction, electrical power, hydraulic power, normalized specific energy consumption, and water flux by osmosis using the calculation methods described in the following section.

### 2.8. Calculation Methods

#### 2.8.1. Electrode Voltage Loss

The voltage loss at the electrodes (including thermodynamic, overpotential, and ohmic contributions) was determined experimentally by measuring the voltage and current density relationship of the electrodes, electrode rinse solution, and a single CEM following Walker et al. [10]. The electrodialysis stack was assembled using a single Neosepta CMB membrane with two end spacers and circulating only electrode rinse solution (i.e., the concentrate and diluate cells were absent). The electrode voltage loss was measured for each current density setpoint, up to 3000 A/m^2^ (300 mA/cm^2^). The three main components of the total electrode voltage loss were modeled following Walker et al. [10]: voltage drop from gas equilibrium at the electrodes (Δϕ_equ_) ≈ 1.23 V, the distance between the electrode and the first membrane of the stack (*w*) = 4.1 mm, conductivity of electrode rinse solution (*κ*_rinse_) = 15.85 mS/cm, the modified transfer coefficient (*α*) = 0.0458, and exchange current density (*i*_o_) = 1.069 A/m^2^. The values of *w*, *α*, and *i*_o_ were determined simultaneously by non-linear regression.

#### 2.8.2. Power and Specific Energy Consumption

Electrical power consumption by the electrodialysis stack was calculated by the multiplication of the applied voltage to the electrodialysis stack after subtracting electrode voltage loss and the electrical current passing through the electrodialysis stack. Energy consumption by the electrodialysis stack was determined by multiplying the consumed electrical power with the experimental period. Energy consumption by the hydraulic pumps was calculated from the flow rate and pressure drop. Moreover, specific energy consumption (SEC) (expressed as kWh/m^3^) was the amount of energy consumed by the desalination process to produce a given volume of product water. Normalized specific energy consumption (nSEC) (expressed as (kWh/m^3^) / (mol/L removed) or (kWh/m^3^) per (eq/L‑removed)) was the amount of energy consumption (kWh) required for the production of one cubic meter (m^3^) product water for per mol/L of salt removal.

The DC electrical power (*P_electrical_*) consumed by the electrodialysis stack was calculated using the formula as below [10]:

*P_electrical_* = Δ*V_stack_ I* (1)

where, Δ*V_stack_* is the voltage drop (V) across the electrodialysis stack after subtracting electrode voltage loss, and *I* is the electrical current (A) measured through the electrodialysis stack.

The hydraulic power (*P_hydraulic_*) for pumping the solution through the electrodialysis stack was calculated using the formula as below [10]:

*P_hydraulic_* = *ρ g Q* Δ*H* (2)

where, ρ is the solution mass-density, g is the gravitational constant, *Q* is the volumetric flow rate, and Δ*H* is the hydraulic head loss through the stack.

The specific energy consumption (SEC) of the batch at time, *t*, was calculated using the formula below [10]:

(3) $$SEC\left(t\right)\;=\;\int_{0}^{t}\frac{P_{\mathrm{electrical}}\left(t\right)\;+\;P_{\mathrm{hydraulic}}\left(t\right)}{Q_{d}}dt$$

where, *Q*_d_ is the volumetric flow rate of the diluate stream.

The normalized SEC was calculated using the formula below [10]:

(4) $$SEC_{\mathrm{normalized}}=\;\frac{SEC}{C_{f}\;-\;C_{d}}$$

where *C_f_* is the concentration of feed solution at the beginning of the experiment (meq/L) and *C_d_* is the concentration (meq/L) of diluate solution at any time (*t*) of the experiment.

#### 2.8.3. Current Density

Electrical current density is the amount of electrical current (or charge flux: Coulombs per second per square meter) passing through the membrane’s active area inside the electrodialysis stack. For a given stack voltage, current density increases with the increase of concentrations in feed solution and the increase of solution velocity in the process streams, whereas current density declines with the decrease of solution temperature in the process streams as the effective cell resistance increases [38,39]. Theoretically, in an ideal electrodialysis system, the ion separation rate is proportional to the electrical current density through the electrodialysis stack. Current density (*i*) was calculated using the following formula [10]:

(5) $$i\;=\frac{I}{A_{mem}}$$

where *I* is the electric current (A) and *A_mem_* is the active area of a single membrane (m^2^).

#### 2.8.4. Limiting Current Density and Limiting Polarization Parameter

The limiting current density (*LCD*) is the maximum allowable current density at which the concentration of salt ions at the membrane surface becomes zero inside diluate cell of electrodialysis stack. Electrodialysis systems should operate at a current density less than the *LCD* in order to prevent water splitting, wastage of power, and damage of electrodialysis equipment. *LCD* depends on the electrodialysis process parameters such as feed water concentration and velocity and temperature of process streams [38,39]. *LCD* increases with the increase of concentration in feed solution and with the increase of solution velocity in the process streams [38,39]. *LCD* declines with the decrease of solution temperature in the process streams, because the effective cell resistance increases [38,39,40].

**Figure 2:**
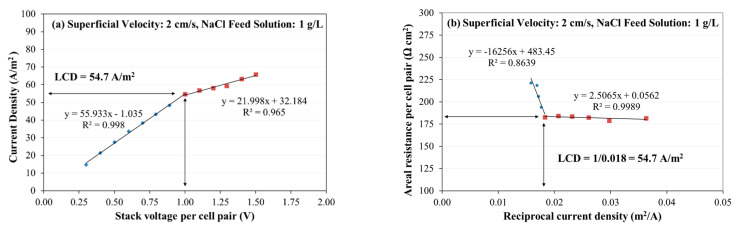
*LCD* determination using (**a**) Shoulder plot and (**b**) Cowan–Brown plot.

*LCD* was determined using the voltage and current data that were recorded during the experiments. Theoretically, there are different approaches employed to determine and compare the *LCD* [41]; firstly, the “Shoulder” method was used, which involves plotting stack voltage on the abscissa and current density (*i*) on the ordinate (Figure 2a), and secondly, the “Cowan–Brown” method was used, which involves plotting inverse of stack’s current density (1/*i*) on the abscissa and stack’s areal electrical resistance per cell-pair on the ordinate (Figure 2b) [39,40,41]. As shown in Figure 2a,b, the *LCD* was the point where two lines (blue and red) intersected in both methods [41].

The limiting polarization parameter (*LPP*) is the limiting current density divided by the normality of the feed solution [10]:

(6) $$LPP\;=\;\frac{LCD}{C_{f}}$$

where, *C_f_* is the concentration (meq/L) of the feed solution. A larger *LPP* value means that the *LCD* is greater for a given feed concentration. If the *LCD* is the “speed limit”, then the the *LPP* is a “normalized speed limit” that is associated with the flow conditions in the diluate cells.

#### 2.8.5. Current Efficiency

Current utilization capacity, known as the current efficiency, Coulombic efficiency, or charge efficiency (*ξ*), is the ratio between the amount of the current used in the electrodialysis stack to effectively separate salt ions (from the diluate to the concentrate stream) and the amount of the total current applied to the electrodialysis stack. Typically, current utilization is greater than 90% in electrodialysis desalination processes [42]. Cumulative charge efficiency (*ξ*) was calculated using the formula below [8,25,36]:

(7) $$\xi \;=\;\frac{\left(C_{f}\;-\;C_{d}\right)V_{d}\;F}{\int IN_{cp}\;dt}$$

where, *C_f_* and *C_d_* are the concentrations (eq/L) of feed solution and diluate solution, respectively, *V_d_* is diulate solution volume (L), F is the Faraday constant (96485.3 Coulombs/eq or Amp-s/eq), *I* is the measured electrodialysis stack current (Amp), and *N*_cp_ is number of cell-pairs in the electrodialysis stack.

#### 2.8.6. Salinity Reduction

Salinity reduction is the ratio of the amount of salt concentration reduction from the initial salt concentration in diluate stream as a function of experimental time. Typical steady-state salinity reduction of a single-pass electrodialysis is in the range of 50% to 60%, depending on source water quality (100–12,000 mg/L TDS), finished water quality (10–1000 mg/L), and system design [42]. Salinity reduction (R) was calculated using the formula below [10]:

(8) $$R\;=\;\frac{\left(C_{f}\;-\;C_{d}\right)}{C_{f}}$$

where *C*_f_ is the concentration of feed solution at the beginning of the experiment and *C*_d_ is the concentration of diluate solution at any time (*t* = 60 min in this study) of the experiment. When applied to desalination with reverse osmosis (RO), Equation (8) is often called “salt rejection”, but that term would be inaccurate if applied to desalination with electrodialysis.

The concentration of sodium chloride was calculated from measured electrical conductivity by the following equation:

(9) $$C\;=\;1.224\times 10^{-9}\;\kappa^{4}\;-\;3.243\times 10^{-7}\;\kappa^{3}\;+\;5.135\times 10^{-5}\;\kappa^{2}\;+\;8.869\times 10^{-5}\;\kappa$$

where *κ* is electrical conductivity in units of mS/cm. This equation is an empirical fit of CRC [43] and Landolt–Börnstein [44] data with relative error less than 1% from 0.1 to 2 mol/L (10.6 to 149 mS/cm), relative error less than 5% from 0.02 to 0.1 mol/L (2.3 to 10.6 mS/cm), and relative error less than 10% from 0.0005 to 0.02 mol/L (0.062 to 2.3 mS/cm).

#### 2.8.7. Water Transport

Water transport through IEMs decreases the efficiency of the ED separation process [3]. Water transport can occur in two different ways such as (i) osmosis water transport (free water or water molecules only) and (ii) electro-osmosis water transport (water bound to ions). Osmosis water transport or flux occurs when only water molecules pass through the membrane due to the larger osmotic pressure differences caused by the difference in concentration of the dilute and concentrate channels. Electro-osmosis water transport occurs when water molecules bound to the primary hydration sphere of the ions pass through the membrane at the same time when ions pass through the membrane [3].

Average water flux by osmosis was calculated using the formula [3,8] below:

(10) $$J_{w}=\;\frac{\Delta m_{w}}{\left(2\;N_{\mathrm{CP}}\;A_{\mathrm{mem}}\right)\;\Delta t_{\mathrm{expt}}}$$

where Δ*m*_w_ is change of mass of water (kg), *N*_cp_ is number of cell-pairs in ED stack (each cell-pair contains two membranes), *A*_mem_ is active area of a single membrane (m^2^), and Δ*t*_expt_ is experiment duration (hr).

## 3. Results

### 3.1. Evaluation of Limiting Current Density and Areal Resistance

**Figure 3:**
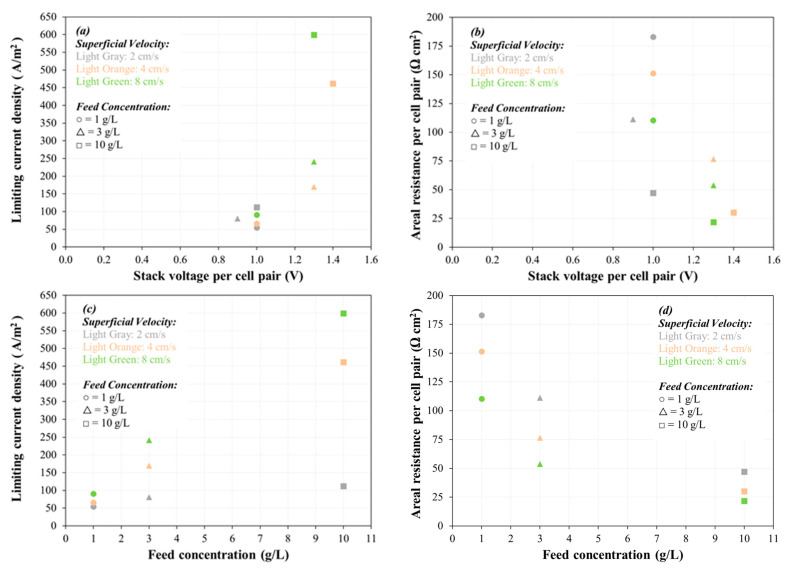

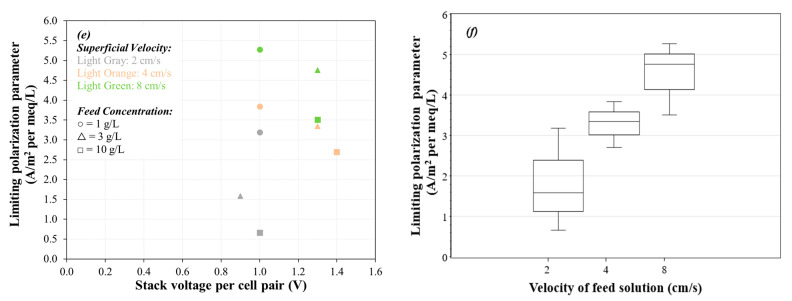
Limiting current density (**a**,**c**), areal resistance (**b**,**d**), and limiting polarization parameter (**e**,**f**). Experimental conditions: Five cell-pairs AMX/CMX stack; 2–8 cm/s superficial velocity; constant stack voltage application; 0.1 M (14.2 g/L) Na_2_SO_4_ electrode rinse solution; and 3 kPa transmembrane pressure.

The limiting current density (*LCD*), the areal resistance per cell-pair of the membrane, and the limiting polarization parameter were identified for feed solution’s superficial velocity of 2, 4, and 8 cm/s at 1, 3, and 10 g/L concentration of NaCl feed solution using Neosepta AMX-CMX ion exchange membrane pair (a well-known commercial membrane), as they were considered as the control membranes in this study (Figure 3). *LCD* and areal resistance results were not achieved for feed solution concentration of 35 and 100 g/L, because the maximum working capacity of the power supply (30 V, 5 A) was reached before observing *LCD* (thus, results are not shown for 35 and 100 g/L feed solution in Figure 3). The *LCD* ranged from 50 to 600 A/m^2^, increasing with salinity and increasing with superficial velocity (Figure 3a,c), which is consistent with other studies [39,40,41]. The voltage application required to achieve *LCD* ranged from 0.9 to 1.4 Volts per cell pair, the corresponding areal resistance per cell pair at *LCD* ranged from 22 to 183 Ω cm^2^ (Figure 3b,d), and the limiting polarization parameter value ranged from 0.66 to 5.28 A/m^2^ per meq/L (Figure 3e). The ranges of the limiting polarization parameter are shown in a quartile box and whisker plot (Figure 3f) for velocity of feed solution of 2, 4, and 8 cm/s. Subsequent experiments were performed with a voltage application less than that observed at *LCD*.

### 3.2. Evaluation of Current Density and Current Efficiency

**Figure 4:**
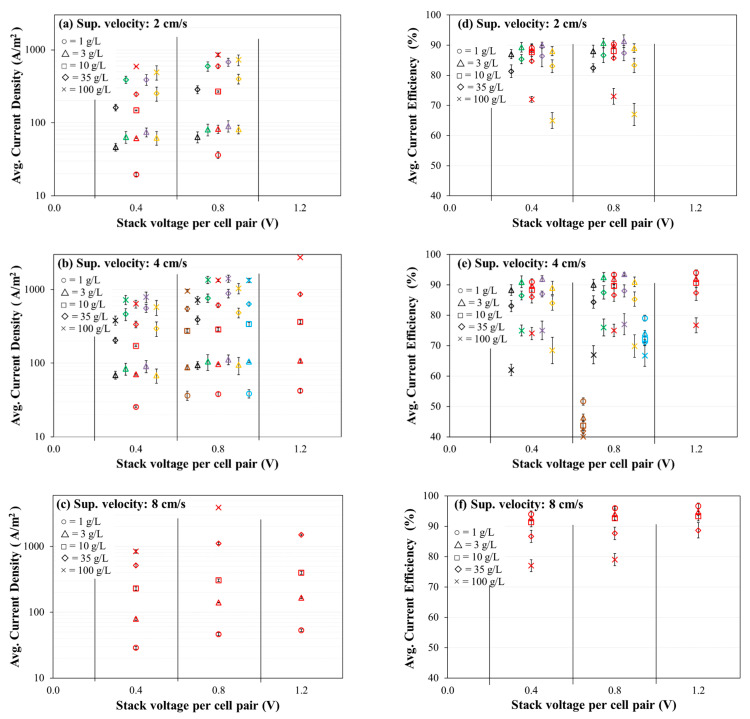
Average current density (**a**–**c**) and average charge efficiency (**d**–**f**) for 60 min of experiment against applied stack voltage per cell pair of the membrane. Experimental conditions: Five cell-pairs stack; 1–100 g/L initial concentration of NaCl feed (diluate and concentrate) solutions (500 mL each); 2, 4, and 8 cm/s superficial velocity of feed solution; 0.4, 0.8, and 1.2 V/cell-pair constant applied stack voltage; 0.1 M (14.2 g/L) Na_2_SO_4_ electrode rinse solution; and 3 kPa transmembrane pressure. Representation of membranes, left to right: Brown: AMX/Polycarb, Black: Ralex CMH-PES/AMH-PES, Green: PCA PCSK/PCSA, Red: Neosepta AMX/CMX, Purple: Fujifilm Type 1 AEM/CEM, Orange: SUEZ AR204/CR67, Blue: AMX/SandiaCEM.

The average current density and the average current efficiency for 60 min of the experimental period were observed for certain permutations of applied stack voltage per cell-pair of membrane, initial concentration of feed solution, and superficial velocity of feed solution for seven combinations of membranes (Figure 4). Note that the abscissa axis (voltage application) is categorical (not linear scale). An increasing trend of average current density was observed with increasing feed salinity for all the membranes (Figure a–c). The average current density for a given membrane and salinity combination increased with increasing stack voltage and increasing velocity. Generally, in Figure 4 parts (a) and (b), the current density for a given feed concentration and voltage application decreased in the following order (i.e., from least to greatest electrical resistance): Fujifilm Type 1 AEM/CEM (purple), PCA PCSK/PCSA (green), AMX/SandiaCEM (blue), Neosepta AMX/CMX (red), SUEZ AR204/CR67 (orange), Ralex CMH-PES/AMH-PES (black), and AMX/Polycarb (brown). The current density was generally negatively correlated with areal resistance (see Table 2).

The average current efficiency for most membranes was greater than 80% for feed salinity of 35 g/L or less, and a decreasing trend of average current efficiency was observed with increasing feed salinity for all the membranes (Figure 4d–f). The average current efficiency for a given membrane and salinity combination increased slightly with increasing stack voltage and increasing velocity. Generally, in Figure 4 parts (d) and (e), the current efficiency for a given feed concentration and voltage application decreased (i.e., from greatest efficiency to least efficiency) in the following order: Fujifilm Type 1 AEM/CEM (purple), PCA PCSK/PCSA (green), Neosepta AMX/CMX (red), SUEZ AR204/CR67 (orange), Ralex CMH-PES/AMH-PES (black), AMX/SandiaCEM (blue), and AMX/Polycarb (brown). As with current density, the current efficiency was generally negatively correlated with areal resistance (see Table 2).

### 3.3. Evaluation of Salinity Reduction and Normalized Specific Energy Consumption

**Figure 5:**
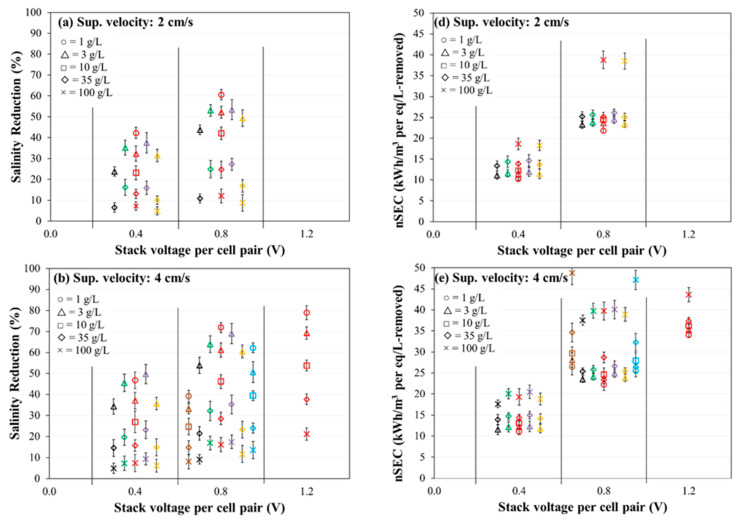

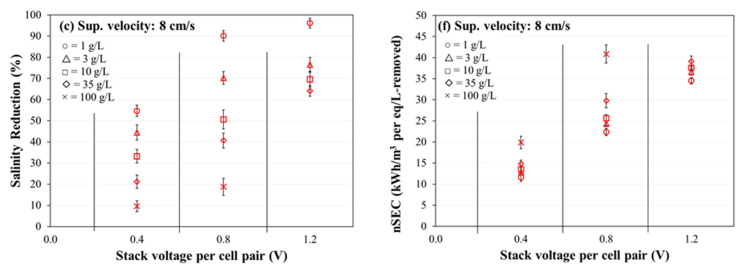
Salinity reduction (**a**–**c**) and normalized specific energy consumption (nSEC) (**d**–**f**) of diluate stream after 60 min of treatment. Experimental conditions: Five cell-pairs stack; 1–100 g/L NaCl initial diluate and concentrate solutions (500 mL each); 2, 4, and 8 cm/s superficial velocity of feed solution; 0.4, 0.8, and 1.2 V/cell-pair constant applied stack voltage; 0.1 M (14.2 g/L) Na_2_SO_4_ electrode rinse solution; and 3 kPa transmembrane pressure. Representation of membranes, left to right: Brown: AMX/Polycarb, Black: Ralex CMH-PES/AMH-PES, Green: PCA PCSK/PCSA, Red: Neosepta AMX/CMX, Purple: Fujifilm Type 1 AEM/CEM, Orange: SUEZ AR204/CR67, Blue: AMX/SandiaCEM.

The salinity (NaCl concentration) reduction in the diluate stream after 60 min of operation was observed for certain permutations of applied stack voltage per cell-pair of membrane, initial concentration of feed solution, and superficial velocity of feed solution for seven combinations of membranes (Figure 5a–c). Note that the abscissa axis (voltage application) is categorical (not linear scale). As expected, a decreasing trend of fractional salinity reduction was observed with increasing feed salinity for all the membranes. The salinity reduction for a given membrane and salinity combination increased significantly with increasing stack voltage and increasing velocity. Generally, in Figure 5 parts (a) and (b), for feed concentrations of 3 and 35 g/L, the salinity reduction for a given voltage application decreased in the following order: Fujifilm Type 1 AEM/CEM (purple), PCA PCSK/PCSA (green), Neosepta AMX/CMX (red), AMX/SandiaCEM (blue), SUEZ AR204/CR67 (orange), Ralex CMH-PES/AMH-PES (black), and AMX/Polycarb (brown). As with current density and current efficiency, salinity removal was generally negatively correlated with areal resistance (see Table 2).

The normalized specific energy consumption (nSEC, energy intensity (kWh/m^3^) per concentration (eq/L) removed) was determined with respect to applied stack voltage per cell-pair of membrane, initial concentration of feed solution, and superficial velocity of feed solution for seven combinations of membranes (Figure 5d–f). Note that the abscissa axis (voltage application) is categorical (not linear scale). An increasing trend of nSEC was observed with increasing feed salinity for all the membranes. The nSEC for a given membrane and salinity combination increased significantly with increasing stack voltage (as expected) and increased slightly with increasing velocity (i.e., the increase in hydraulic pumping power with increasing velocity outweighs the decrease in resistances of the diffusion boundary layers). Generally, in Figure 5 parts (d) and (e), the nSEC for a given feed concentration (3 and 35 g/L) and voltage application increased in the following order (i.e., from least energy demand to greatest energy demand): Ralex CMH-PES/AMH-PES (black), SUEZ AR204/CR67 (orange), Neosepta AMX/CMX (red), PCA PCSK/PCSA (green), Fujifilm Type 1 AEM/CEM (purple), AMX/SandiaCEM (blue), and AMX/Polycarb (brown). The nSEC increases with the increase in electrical resistance of IEMs.

For feed concentrations in the range of 3 to 35 g/L, and a voltage application of 0.8 volts per cell pair, most of the membranes had very similar normalized energy consumption in the range of 23 to 27 kWh/m^3^ per meq/L removed. The Ralex CMH-PES/AMH-PES (black) membranes were on the lower end of salinity reduction and normalized energy consumption in comparison with the other membranes. The AMX/SandiaCEM (blue) and AMX/Polycarb (brown) membranes were generally on the lower end of salinity reduction and higher end of normalized energy consumption, which shows opportunities for improving the permselectivity of the membranes.

### 3.4. Evaluation of Water Flux by Osmosis

**Figure 6:**
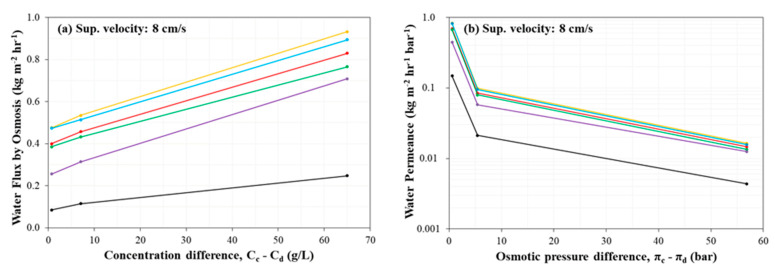
Water flux by osmosis versus concentration difference (**a**) and osmotic permeance versus osmotic pressure difference (**b**). Experimental conditions: Five cell-pairs stack; 0.7, 7, and 65 g/L initial concentration differences between NaCl concentrate and diluate streams (500 mL each) corresponding to 0.6, 5.4, and 56.1 atm osmotic pressure differences between NaCl concentrate and diluate stream, respectively; 8 cm/s superficial velocity of feed solution; no applied stack voltage per cell-pair; 0.1 M (14.2 g/L) Na_2_SO_4_ electrode rinse solution; and 3 kPa transmembrane pressure. Representation of membranes, top to bottom: Orange: SUEZ AR204/CR67, Blue: AMX/SandiaCEM, Red: Neosepta AMX/CMX, Green: PCA PCSK/PCSA, Purple: Fujifilm Type 1 AEM/CEM, Black: Ralex CMH-PES/AMH-PES.

The water flux and permeance due to osmosis was measured with respect to the concentration differences and the osmotic pressure difference between concentrate and diluate streams for six combinations of membranes (Figure 6a,b). The osmotic water flux experiments were performed at the highest superficial velocity of feed solution of 8 cm/s without the application of any stack voltage (tests at 2 and 4 cm/s were omitted because greater osmotic water flux is achievable at the higher superficial velocity). The initial concentration differences between concentrate and diluate were 0.7 g/L (1 vs. 0.3 g/L), 7 g/L (10 vs. 3 g/L), and 65 g/L (100 vs. 35 g/L), which corresponded to osmotic pressure differences of 0.58, 5.42, and 56.81 bar, respectively.

Generally, in Figure 6, the osmotic water flux increased in the following order (i.e., from least water flux to greatest water flux): Ralex CMH-PES/AMH-PES (black), Fujifilm Type 1 AEM/CEM (purple), PCA PCSK/PCSA (green), Neosepta AMX/CMX (red), AMX/SandiaCEM (Blue), and SUEZ AR204/CR67 (orange) (Figure 6a,b). The Ralex (CMH-PES/AMH-PES) membrane pair exhibited a much lower osmotic water flux than the other four membranes, which was expected. As a heterogeneous membrane, Ralex (CMH-PES/AMH-PES) carries the non-uniform distribution of water content, crosslinking reagent, and charge density in its morphological structure; these properties consequently cause the higher resistance to permeation compared to other four homogeneous commercial IEMs. Other than the Ralex membranes, osmotic permeance was not well correlated with membrane thickness, ion exchange capacity, or areal electrical resistance. For comparison, water permeance in ion exchange membranes is also reported in Kingsbury et al. [45].

## 4. Conclusions and Recommendations

Laboratory-scale batch-recycle electrodialysis desalination experiments with aqueous sodium chloride solutions ranging from 1 to 100 g/L were performed with permutations of voltage application (0.4, 0.8, and 1.2 V per cell pair) and superficial feed velocity (2, 4, and 8 cm/s) to compare five commercial ion exchange membrane sets with a novel bioinspired cation exchange membrane developed recently at Sandia National Labs [13,14]. The significant conclusions of the study are summarized below:

1. The limiting current density (*LCD*) of an ED stack with Neosepta AMX/CMX membranes, feed solution of 1 to 10 g/L, and superficial velocity of 2 to 8 cm/s ranged from 50 to 600 A/m^2^, increasing with salinity and increasing with superficial velocity. The voltage application required to achieve *LCD* ranged from 0.9 to 1.4 Volts per cell pair, and the corresponding areal resistance per cell pair at *LCD* ranged from 22 to 183 Ω cm^2^. The limiting polarization parameter ranged from 0.66 to 5.3 A/m^2^ per meq/L.
2. Average current efficiency was observed to decrease with increasing feed salinity for all the membranes. The average current efficiency for a given membrane and salinity combination increased slightly with increasing stack voltage and increasing velocity. Generally, for a given feed concentration and voltage application the current efficiency decreased in the following order (i.e., from greatest efficiency to least efficiency): Fujifilm Type 1 AEM/CEM, PCA PCSK/PCSA, Neosepta AMX/CMX, SUEZ AR204/CR67, Ralex CMH-PES/AMH-PES, AMX/SandiaCEM, and AMX/Polycarb.
3. The fractional salinity reduction was observed to decrease with increasing feed salinity for all the membranes, but for a given membrane and feed salinity, the salinity reduction increased significantly with increasing stack voltage and increasing velocity. Generally, the Ralex CMH‑PES/AMH-PES, AMX/SandiaCEM, and AMX/Polycarb membranes were on the lower end of salinity reduction, and Fujifilm Type 1 AEM/CEM showed the greatest salinity reduction for a given feed concentration (3 and 35 g/L) and voltage application. The rest of the membranes showed quite similar performance in salinity reduction, with slightly more differentiation at lower feed concentrations.
4. The normalized specific energy consumption (nSEC, kWh/m^3^ per eq/L removed) was observed to increase with increasing feed salinity for all the membranes. The nSEC for a given membrane and salinity combination increased significantly with increasing stack voltage and increased slightly with increasing velocity. Generally, the Ralex CMH-PES/AMH-PES membranes consumed the least energy but AMX/SandiaCEM and AMX/Polycarb membranes were on the higher end of energy consumption compared to the other membranes for a given feed concentration (3 and 35 g/L) and voltage application. The rest of the membranes showed quite similar performance from a nSEC perspective, with slightly more differentiation at higher feed concentration.
5. Water flux by osmosis was observed to increase with the increase of concentration difference (i.e., osmotic pressure difference for a given IEM and superficial velocity). Generally, the osmotic water flux increased in the following order (i.e., from least osmotic flux to greatest osmotic flux): Ralex CMH-PES/AMH-PES (black), Fujifilm Type 1 AEM/CEM (purple), PCA PCSK/PCSA (green), Neosepta AMX/CMX (red), AMX/SandiaCEM (Blue), and SUEZ AR204/CR67.
6. The ED desalination performance of the Sandia novel bioinspired cation exchange membrane (SandiaCEM) was observed to be competitive with the commercial cation exchange membranes.

For the sake of simplicity, a membrane performance comparison is provided in Table 3. For most desalination applications in which the total life cycle costs are strongly influenced by energy costs, it is desirable to use membranes with a lower areal resistance and higher current efficiency; in high-salinity applications, it is very important to select low-resistance membranes, but there are some fresh/brackish applications in which the electrical resistance of the diluate cells greatly outweighs the membrane resistance. For desalination applications targeting a very high water recovery, a low water permeance is a key membrane selection parameter.

Future work should investigate the long-term mechanical stability and durability of the novel bioinspired membrane.

## Figures and Tables

**Table 1:** Experimental variables, value ranges, and combinations.

Variables	Discrete Values/Combinations
NaCl feed water concentration	1, 3, 10, 35, 100 g/L
Superficial velocity of diluate stream	2, 4, 8 cm/s (corresponding flow: 15, 30, 60 mL/min)
Stack voltage	0.4, 0.8, 1.2 V/cell-pair
Combination of membranes during stack assembly	i. Neosepta AMX & CMX ii. PCA PCSA & PCSK iii. Fujifilm Type 1 AEM & CEM iv. SUEZ AR204SZRA & CR67HMR v. Ralex AMH-PES & CMH-PES vi. Neosepta AMX & bare polycarbonate (Polycarb) vii. Neosepta AMX & Sandia novel bioinspired CEM (SandiaCEM)

**Table 2:** Standard properties of IEMs used in this study [15,16,17,18,19,20,21,22,23,24,25,26].

Membrane	Type	Thickness (mm)	IEC (meq/g)	Areal Resistance (Ω cm^2^)	Remarks	Ref.
Polycarbonate	-	0.006	-	10.3	Filtering air/water	[22]
Ralex AMH-PES *	AEM	0.55 Dry	1.8	<8	ED, EDI	[15,20]
Ralex CMH-PES *	CEM	0.45 Dry	2.2	<9	ED, EDI	[15,20]
PCA PCSA	AEM	0.232	1.69	-	Standard ED	[19]
PCA PCSK	CEM	0.098	1.25	-	Standard ED	[19]
Neosepta AMX	AEM	0.12–0.18	1.4–1.7	2.0–3.5	High strength	[15,17]
Neosepta CMX	CEM	0.14–0.20	1.5–1.8	2.0–3.5	High strength	[15,17]
Fujifilm Type 1 AEM	AEM	0.125	1.50	1.3	Water softening	[21]
Fujifilm Type 1 CEM	CEM	0.135	1.43	2.7	Water softening	[21]
SUEZ AR204SZRA	AEM	0.48–0.66	2.3–2.7	6.2–9.3	EDR	[15,20]
SUEZ CR67HMR	CEM	0.53–0.65	2.1–2.45	7.0–11.0	ED	[15,20]
Sandia CEM *	CEM	0.0072	-	18.5	ED, EDR	[13]

Note: * Heterogeneous membranes (others are homogeneous), IEC: ion-exchange capacity, ED: electrodialysis, EDR: electrodialysis reversal, and EDI: electro-deionization. Each color represents a specific membrane combination, consistent with the graphical abstract and other figures in this manuscript.

**Table 3:** Membrane performance comparison.

Membrane	Ralex CMH-PES/AMH-PES	PCA PCSK/PCSA	Neosepta AMX/CMX	Fujifilm Type 1 AEM/CEM	SUEZ AR204/CR67	AMX/SandiaCEM
Current Density *	min	>med	>med	**max**	<med	>med
Current Efficiency *	<med	>med	>med	**max**	<med	min
Salinity Reduction	min	>med	>med	**max**	<med	<med
Normalized SEC	**min**	>med	>med	>med	<med	max
Water Permeance	**min**	<med	>med	<med	max	>med

Notes: * for 0.8 V/cell-pair, 4 cm/s, and 3 to 35 g/L; “min” minimum of the six membranes; “<med” less than median; “>med” greater than median; “max” maximum; **boldface** indicates generally preferred attribute, each color represents a specific membrane combination, consistent throughout the tables and figures in this manuscript.

## Data Availability

The data presented in this study are available on request from the corresponding author.
